# Identification of Genes Encoding Granule-Bound Starch Synthase Involved in Amylose Metabolism in Banana Fruit

**DOI:** 10.1371/journal.pone.0088077

**Published:** 2014-02-04

**Authors:** Hongxia Miao, Peiguang Sun, Weixin Liu, Biyu Xu, Zhiqiang Jin

**Affiliations:** 1 Key Laboratory of Tropical Crop Biotechnology, Ministry of Agriculture, Institute of Tropical Bioscience and Biotechnology, China Academy of Tropical Agricultural Sciences, Haikou, Hainan, China; 2 Key Laboratory of Genetic Improvement of Bananas, Hainan Province, Haikou Experimental Station, China Academy of Tropical Agricultural Sciences, Haikou, Hainan, China; 3 Department of Agriculture, Hainan University, Haikou, Hainan, China; Institute of Botany, Chinese Academy of Sciences, China

## Abstract

Granule-bound starch synthase (GBSS) is responsible for amylose synthesis, but the role of *GBSS* genes and their encoded proteins remains poorly understood in banana. In this study, amylose content and GBSS activity gradually increased during development of the banana fruit, and decreased during storage of the mature fruit. GBSS protein in banana starch granules was approximately 55.0 kDa. The protein was up-regulated expression during development while it was down-regulated expression during storage. Six genes, designated as *MaGBSSI-1*, *MaGBSSI-2*, *MaGBSSI-3*, *MaGBSSI-4*, *MaGBSSII-1*, and *MaGBSSII-2*, were cloned and characterized from banana fruit. Among the six genes, the expression pattern of *MaGBSSI-3* was the most consistent with the changes in amylose content, GBSS enzyme activity, GBSS protein levels, and the quantity or size of starch granules in banana fruit. These results suggest that *MaGBSSI-3* might regulate amylose metabolism by affecting the variation of GBSS levels and the quantity or size of starch granules in banana fruit during development or storage.

## Introduction

Starch is the main carbohydrate consumed for human nutrition, and is a major component of cereals, tubers, legumes, and fruits. Starch consists of a mixture of two different carbohydrates, namely amylose (20%–30%) and amylopectin (70%–80%) [1]–[3]. Amylose is a linear polymer of glucose residues joined together by α-1, 4-glucosidic bonds, and its synthesis is mainly catalyzed through the activity of granule-bound starch synthase (GBSS). GBSS transfers the glucosyl residue from ADP-Glu to glucan substrates to produce relatively long-chain amylose molecules [4]. The amylose content directly affects the texture and taste of cereals grains [5]. The physical structure of amylopectin has an important impact on the crystalline properties of maize [5], and its synthesis requires soluble starch synthases (SSs), starch branching enzymes (SBEs), and starch debranching enzymes (DBEs) [4].

GBSS proteins are crucial in regulating the formation of amylose. Inhibition of GBSSI expression by RNAi interference resulted in an amylose-free transgenic sweet potato [6]. GBSS activity and amylose content also decreased significantly after RNA silencing of *GBSSI* gene expression in the endosperm of wheat [7]. A full-length sense cDNA encoding sweet potato *GBSSI*, driven by the CaMV 35S promoter, was introduced into sweet potato by *Agrobacterium tumefaciens*-mediated transformation. One of the resulting 26 transgenic plants had an absence of amylose in the tuberous roots [8]. Recently, through regulated *GBSS* gene expression in sweet potato, starch has been produced with varying amylose-amylopectin ratios. Low-amylose starch may be used in the food industry, while high-amylose starch is useful in the candy industry and for synthesizing plastics [9].

GBSS proteins are also known as waxy proteins [10]. In monocots, GBSS is divided into two families, i.e. GBSSI and GBSSII. GBSSI transcripts are predominantly found in endosperm, embryos and pollen, while GBSSII transcripts are expressed in non-storage tissues such as leaves, stems, roots, and other organs [11]. In contrast, all *GBSS* genes in eudicots belong to the GBSSII family and their expression was consistent with the pattern observed for *GBSSII* genes in monocots. This suggests that the GBSSI subfamily has been lost during the evolutionary process leading to eudicots [1]. To date, the sequence and expression of *GBSSI* genes has been characterized from maize [12], rice [13], barley [5], wheat [11], pea [14], potato [15], and sweet potato [8]. *GBSSII* genes were isolated from rice [13], wheat [11], apple, peach, and orange [1]. However, the complete sequence and expression patterns of these genes from banana fruit have not been reported yet. To facilitate further studies of banana fruit starch, it is essential to characterize the banana *GBSSI* and *GBSSII* genes in banana.

Banana (*Musa* spp.) is the main staple food of the tropics. Its fruits are vital for food security in many tropical and subtropical counties and banana is also among the most popular fruits in industrialized countries [16]–[17]. Starch is the principal component of green banana fruit and is present at levels of approximately 61.3–76.5%, which is sufficient for industrial-scale purification of starch [12]. Recently, a complete genome sequence has been released for banana (http://banana-genome.cirad.fr/). This genome sequence database provides unique opportunities for the genome-wide investigation of genes involved in banana fruit starch synthesis. In this study, we investigated the changes in amylose content, GBSS enzyme activity and GBSS protein from preharvest to postharvest of banana (*Musa acuminata* L. AAA group cv. Brazilian) fruit. Six *MaGBSS* genes were cloned, and their sequence characteristics, chromosomal location, and expression patterns were studied in different tissues and at different stages of fruit development or storage. The number, size, and shape of starch granules in banana fruit during development and storage were also observed.

## Results

### Changes in total starch content, amylose content and GBSS activity

**Figure 1:**
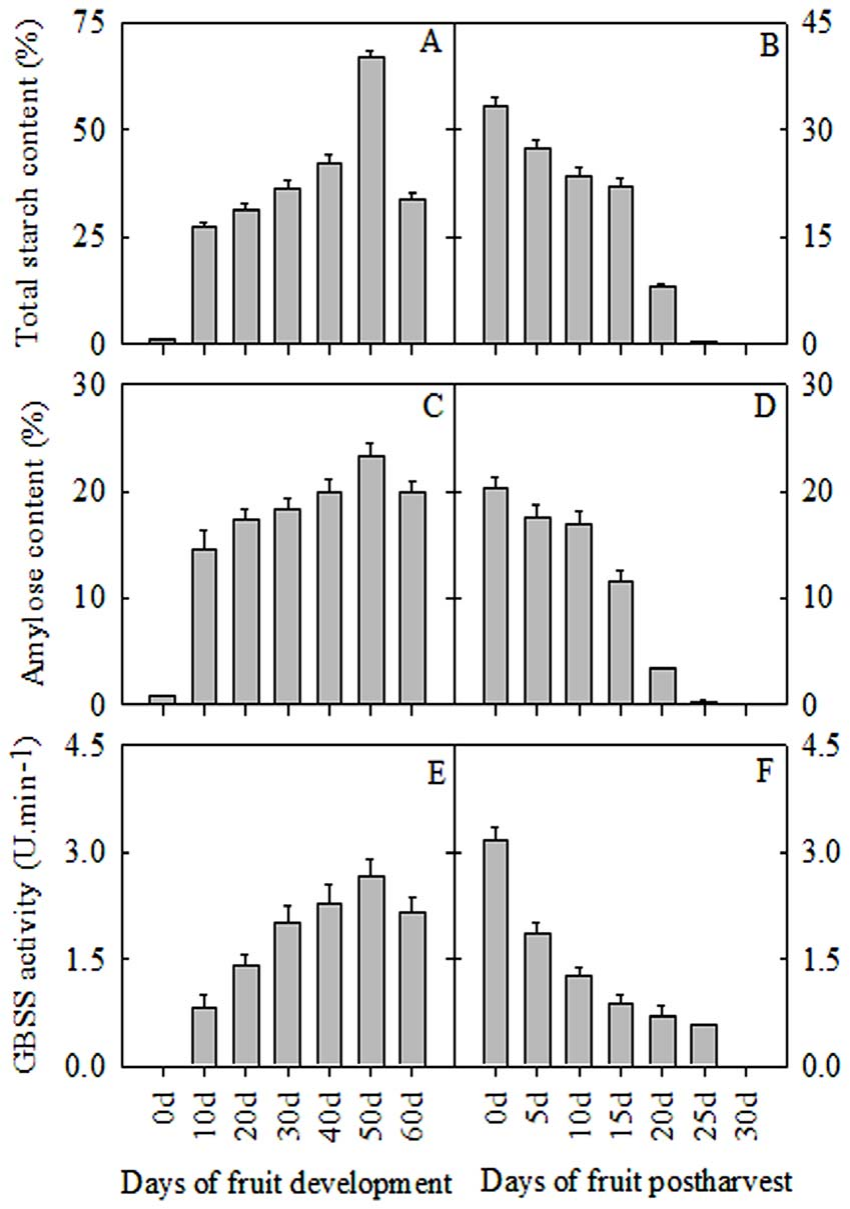
Changes in total starch content (A, B), amylose content (C, D), and GBSS activity (E, F) in banana pulp at different stages of development and storage. The vertical bars represent standard error (±SE) of three replicates. Three biological experiments were performed, which produced similar results.

Total starch content increased gradually during the development of banana fruit but decreased after harvest (Fig. 1A and 1B). Pulp amylose content increased from 0 d to 50 d of fruit development and declined at the 60 d time point. The amylose content also decreased gradually from 0 d to 30 d of storage (Fig. 1C and 1D). An increase in GBSS activity occurred from 0 d to 50 d of development but declined at 60 d, while GBSS activity decreased sharply from 0 d to 30 d of storage (Fig. 1E and 1F).

### SDS-PAGE and western blotting analysis of GBSS protein

**Figure 2:**
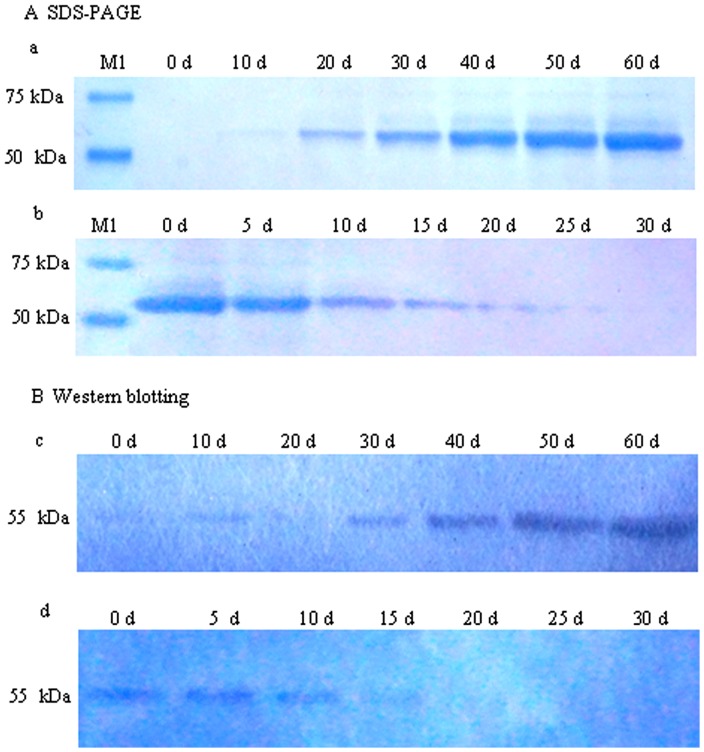
SDS-PAGE and western blotting analyses of GBSS proteins in banana pulp at different stages of development and ripening. M1: Protein marker; A: SDS-PAGE; B: Western blotting; a: Variation of GBSS protein levels at different development stages of banana fruit by SDS-PAGE analysis; b: Variation of GBSS protein levels at different ripening stages of banana fruit by SDS-PAGE analysis; c: Variation of GBSS protein levels at different development stages of banana fruit by western blotting analysis; d: Variation of GBSS protein levels at different ripening stages of banana fruit by western blotting analysis. Each lane contained 20 µL of the GBSS protein extract.

To determine whether banana fruit starch contains GBSS protein, starch granules in banana pulp were isolated; GBSS protein was purified and analyzed on SDS-PAGE gels (Fig. 2A). The 55.0 kDa GBSS protein was migrated in the pulps by western blotting analyses (Fig.2B). The expression of GBSS protein was gradually up-regulated during the development of banana fruit, but a decrease was observed from 0 d to 30 d of storage (Fig. 2B). These SDS-PAGE and western blotting results are consistent with the changes in amylose content (Fig. 1C and 1D) and GBSS activity (Fig. 1E and 1F) during banana development and storage.

### Nucleotide sequence characteristics and chromosomal localization of six *GBSS* genes in banana

Six genes encoding *GBSS* family members, designated as *MaGBSSI-1*, *MaGBSSI-2*, *MaGBSSI-3*, *MaGBSSI-4*, *MaGBSSII-1*, and *MaGBSSII-2*, were cloned from banana. Full-length cDNAs encoding *MaGBSSI-1*, *MaGBSSI-2*, *MaGBSSI-3*, *MaGBSSI-4*, *MaGBSSII-1*, and *MaGBSSII-2* were 1851 bp (accession No. KF512020), 1851 bp (accession No. KF512021), 675 bp (accession No. KF512022), 1845 bp (accession No. KF512023), 2265 bp (accession No. KF512024), and 906 bp (accession No. KF512025), respectively. Genes encoding *MaGBSSI-1*, *MaGBSSI-2*, and *MaGBSSI-4* contain 13 exons and use TGA as stop codon. *MaGBSSI-3* consists of 6 exons and uses TAG as stop codon. *MaGBSSII-1* and *MaGBSSII-2* contain 10 exons and 1 exon, respectively, and use TAA as stop codon (Fig. S1). The six genes encoding *MaGBSS* family members were BLASTed against the banana genome sequence database (http://banana-genome.cirad.fr/). *MaGBSSI-1*, *MaGBSSI-2*, and *MaGBSSI-3* are dispersed along chromosome 3. *MaGBSSII-1*, *MaGBSSII-2*, and *MaGBSSI-4* are located on chromosomes 4, 8, and 9, respectively (Fig. S1).

### Amino acid sequence analysis of six *MaGBSS* genes

The deduced amino acid sequences for the cDNA of the six MaGBSS proteins in banana shared three conserved regions, referred to as Box1, Box2, and Box3 (Fig. S2). Compared with the MaGBSSI-1, MaGBSSI-2, MaGBSSI-4, and MaGBSSII-1 amino acid sequences, there were 14 amino acids (PWSKTGGLGDVLGGLP) absent in Box 1 of MaGBSSI-3 and MaGBSSII-2. With respect to the Box 2 (TSRFEPCGL) sequence of MaGBSSI-1, MaGBSSI-2, and MaGBSSI-4, there were 5 amino acid differences (T→S, F→M, P→F, C→Q, G→N) in the Box 2 of MaGBSSI-3 (Fig. S2). The predicted molecular weights of MaGBSSI-1, MaGBSSI-2, MaGBSSI-3, MaGBSSI-4, MaGBSSII-1, and MaGBSSII-2 peptides were 67.7 kDa, 68.0 kDa, 55.6 kDa, 67.7 kDa, 83.6 kDa, and 34.0 kDa, respectively. The MaGBSSI-3 protein was 55.6 kDa according to pI/MW software analysis (http://expasy.org/compute.pi/), which was consistent with the GBSS protein detected using SDS-PAGE and western blotting analyses (Fig. 2A and 2B).

**Figure 3:**
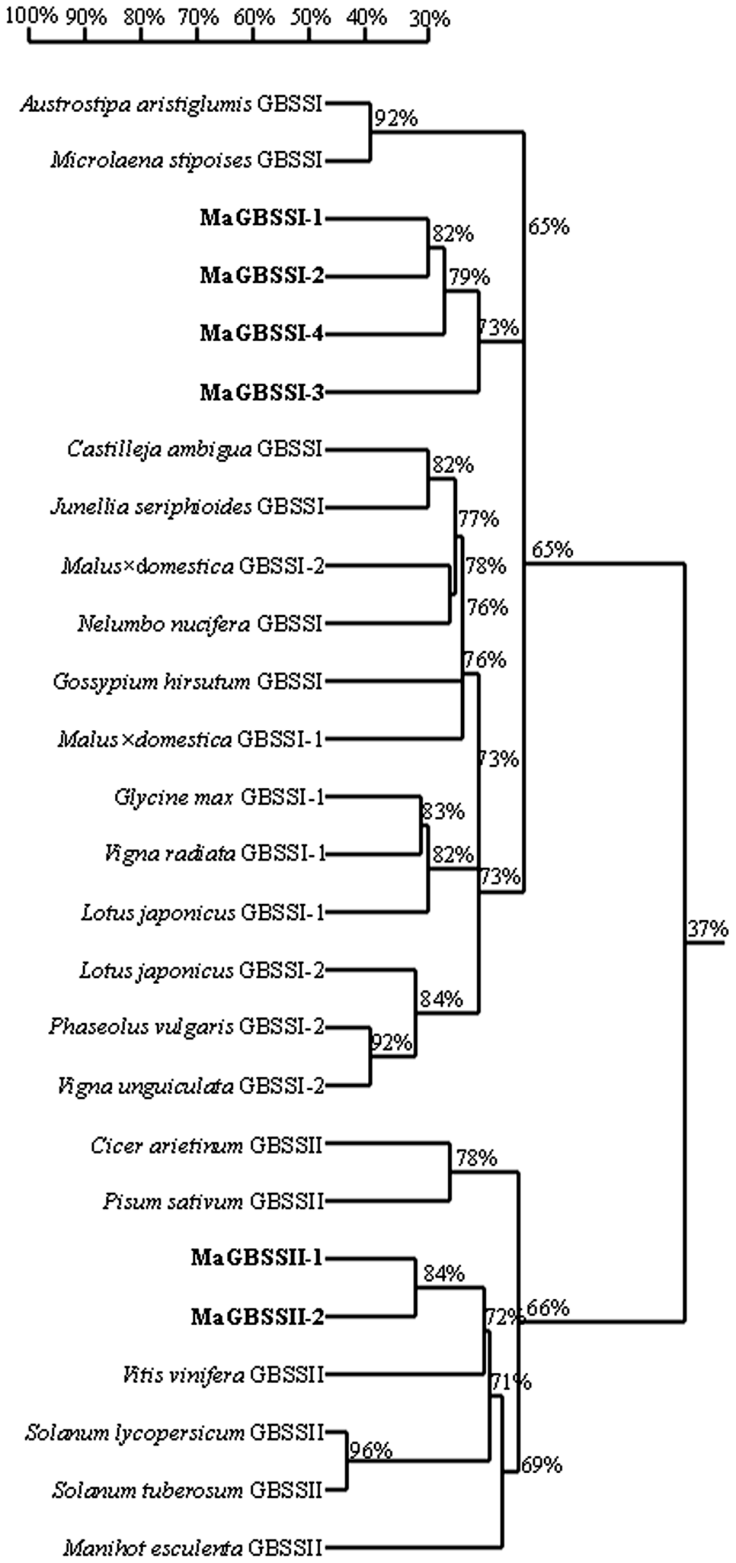
Phylogeny of MaGBSSI and MaGBSSII amino acid sequences. *Austrostipa aristiglumis* GBSSI (ABU98330), *Microlaena stipoises* GBSSI (ABU99332), MaGBSSI-1, MaGBSSI-2, MaGBSSI-3, and MaGBSSI-4 (*Musa acuminate* L. AAA group cv. Brazilian), *Castilleja ambigua* GBSSI (ACZ73348), *Junellia seriphioides* GBSSI (ABQ52190), *Malus×domestica* GBSSI-2 (ACB97678), *Nelumbo nucifera* GBSSI (ACM78591), *Gossypium hirsutum* GBSSI (ACV72639), *Malus×domestica* GBSSI-1 (ACB97677), *Glycine max* GBSSI-1 (NP001237971), *Vigna unguiculata* GBSSI-1 (ABP35818), *Lotus japonicus* GBSSI-1 (ACB30384), *Lotus japonicus* GBSSI-2 (ACB30385), *Phaseolus vulgaris* GBSSI-2 (BAC76613), *Vigna radiata* GBSSI-2 (ACB30382), *Cicer arietinum* GBSSII (XP004489397), *Pisum sativum* GBSSII (Q43093), MaGBSSII-1 and MaGBSSII-2 (*Musa acuminate* L. AAA group cv. Brazilian), *Vitis vinifera* GBSSII (XP002278470), *Solanum lycopersicum* GBSSII (XP004232219), *Solanum tuberosum* GBSSII (Q43847), *Manihot esculenta* GBSSII (AAF13168). Numbers presented as percentages indicate the identity of MaGBSSI and MaGBSSII amino acid sequences from different species.

Amino acid sequence analysis showed that the MaGBSSI-1 had the closest relationship with MaGBSSI-2 and shared 82% amino acid identity, followed by MaGBSSI-4 and MaGBSSI-3 (Fig. 3). MaGBSSII-1 had the closest relationship with MaGBSSII-2 and shared 84% amino acid identity, followed by *Vitis vinifera* (XP002278470) (Fig. 3). A phylogenetic tree showed that six MaGBSS amino acid sequences from banana fruit are very conservative and share high sequence similarity with other plant species (Fig. 3).

### Expression of *MaGBSSI* and *MaGBSSII* genes in banana tissues

**Figure 4:**
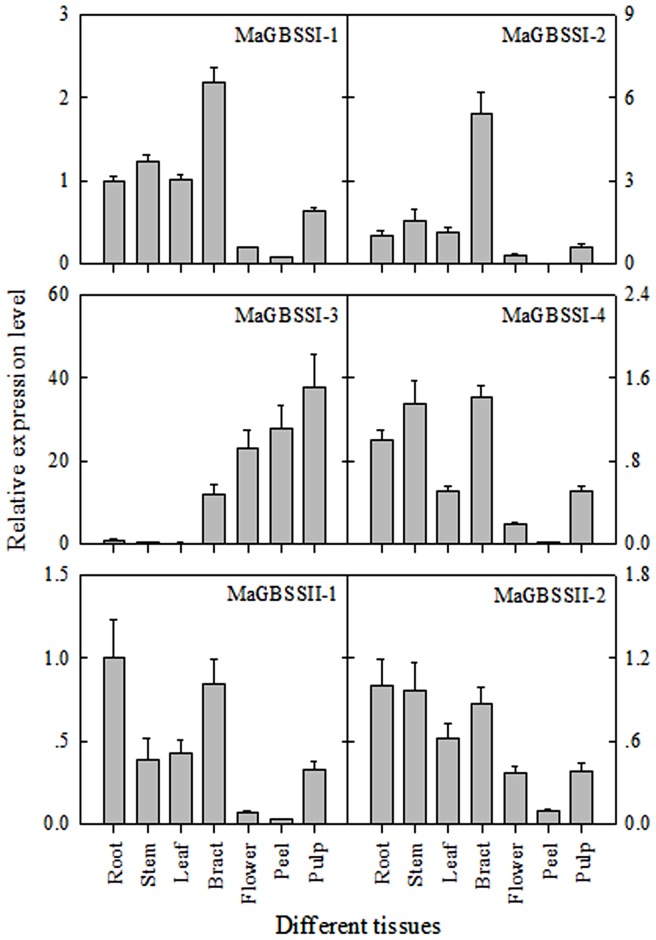
Expression of *MaGBSSI* and *MaGBSSII* genes in different banana tissues. The y-axis represents the relative fold difference in mRNA level, which is calculated using the 2^−ΔΔCt^ formula with *MaActin* as internal control. Relative expression levels are presented as fold-changes relative to the expression level obtained in root tissue. The vertical bars represent standard error (±SE) of three replicates. Three biological experiments were performed, which produced similar results.

Q-RTPCR revealed significant differences in the expression of *MaGBSSI* and *MaGBSSII* in different banana tissues (Fig. 4). *MaGBSSI-1*, *MaGBSSI-2*, *MaGBSSI-4*, *MaGBSSII-1*, and *MaGBSSII-2* were upregulated in vegetative tissue such as root, stem, leaf, and bract. In contrast, *MaGBSSI-3* was highly expressed in reproductive tissues such as flower, peel, and pulp, but was weakly expressed in root, stem, and leaf. These results suggest that *MaGBSSI-3* might be involved in amylose metabolism in reproductive tissues.

### Expression of six *MaGBSS* genes and scanning electron microscopy (SEM) of starch granules at different development stages of banana fruit

**Figure 5:**
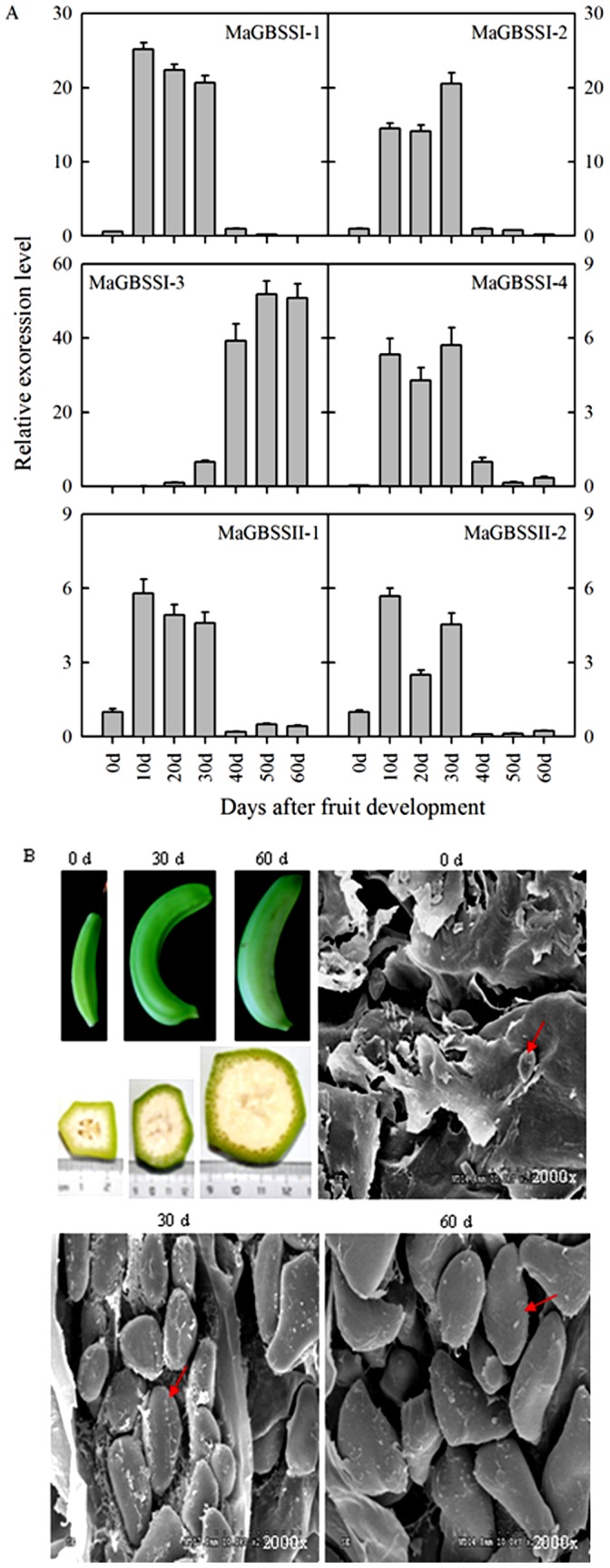
Expression of *MaGBSS* genes (A) and scanning electron microscopy (SEM) of starch granules (B) at different development stages in banana fruit. The y-axis represents the relative fold difference in mRNA level, which is calculated using the 2^−ΔΔCt^ formula with *MaActin* as internal control. Relative expression levels are presented as fold-changes relative to the expression level obtained at 0 day of fruit development. The vertical bars represent standard error (±SE) of three replicates (A). Red arrow represents the starch granules. Three biological experiments were performed, which produced similar results.

The expression of *MaGBSSI* and *MaGBSSII* genes at different development stages of banana fruit was determined by Q-RTPCR. Expression levels of *MaGBSSI-1*, *MaGBSSI-2*, *MaGBSSI-4*, *MaGBSSII-1*, and *MaGBSSII-2* at earlier stages of banana development (from 0 d to 30 d) were higher than the later stages (from 30 d to 60 d). In contrast, *MaGBSSI-3* was weakly expressed at the early stages but was highly upregulated (approximately 50-fold) at 50 d of development (Fig. 5A). These results suggest that the *MaGBSSI-1*, *MaGBSSI-2*, *MaGBSSI-4*, *MaGBSSII-1*, and *MaGBSSII-2* might be involved in the early stages (0–30 d) of starch granule-filling, and *MaGBSSI-3* might be involved in the later stages (30–60 d) of starch granule-filling during the development of banana fruit (Fig. 5A).

Starch granules within banana fruit at 0 d, 30 d, and 60 d of fruit development were observed using SEM. Granules could scarcely be observed at 0 d, but the number and size of oval-shaped granules significantly increased at 30 d of development. In comparison to the granules at 30 d, the number and shape of granules at 60 d was similar, but they were significantly larger (Fig. 5B). These results suggest that the first 30 d of fruit development may be critical for settling the final number and shape of starch granules in banana fruit. 30–60 d of fruit development appears to be the rapid filling phase of starch granules. These results are consistent with the expression profile of *MaGBSSI-3* during the development of banana fruit.

### Expression of six *MaGBSS* genes and scanning electron microscopy (SEM) of starch granules following storage of mature banana fruit

**Figure 6:**
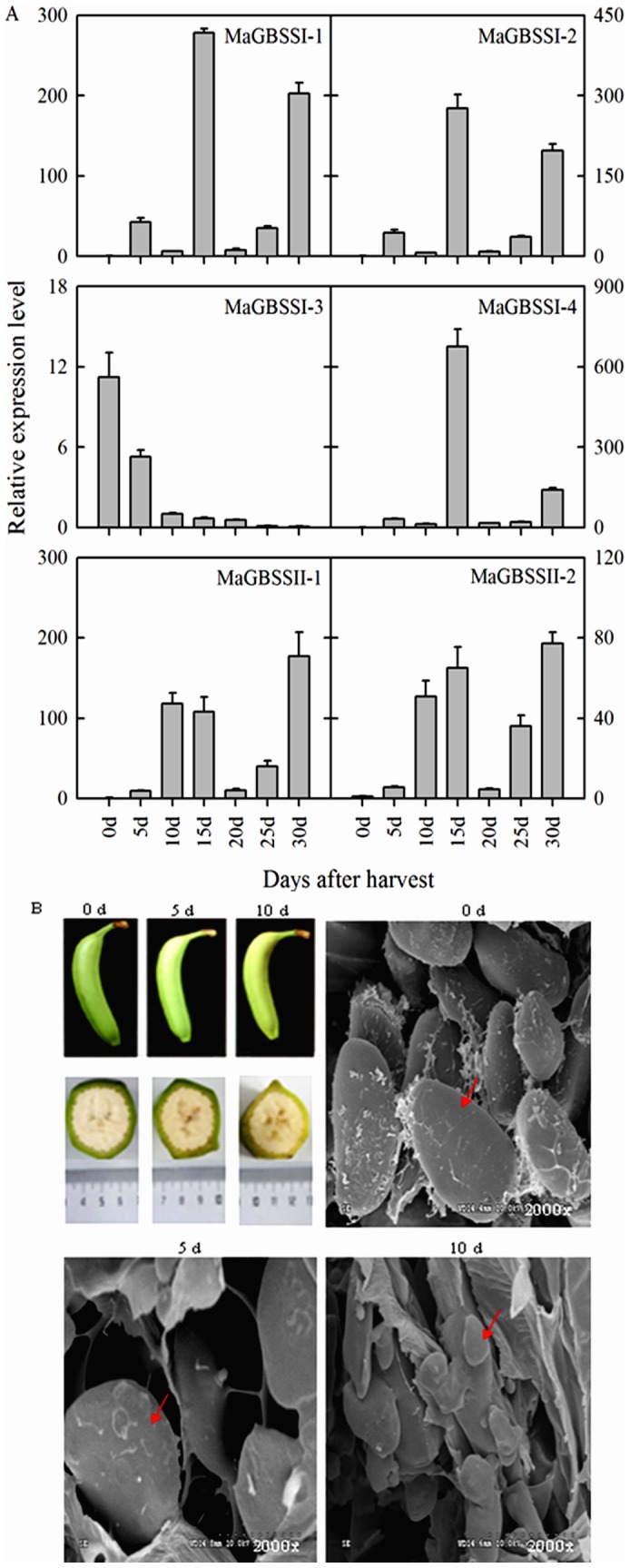
Expression of *MaGBSS* genes (A) and scanning electron microscopy (SEM) of starch granules (B) in banana fruit stored for various periods of time. The y-axis represents the relative fold difference in mRNA level, which is calculated using the 2^−ΔΔCt^ formula with *MaActin* as internal control. Relative expression levels are presented as fold-changes relative to the expression level obtained at 0 day of fruit postharvest. The vertical bars represent standard error (±SE) of three replicates (A). Red arrow represents the starch granules. Three biological experiments were performed, which produced similar results.

Similar expression profiles of *MaGBSSI-1*, *MaGBSSI-2*, *MaGBSSI-4, MaGBSSII-1,* and *MaGBSSII-2* were detected in naturally ripened banana fruits during different storage time. Their expression increased from 0 d to 15 d of storage, declined at the 20 d time point, and then increased again until 30 d of storage. In contrast, a continuous decrease in *MaGBSSI-3* expression occurred from 0 d to 30 d of storage. The gene was abundantly expressed only in the early stages (from 0 d to 10 d) of fruit ripening and was barely detectable during the later stages (10 d–30 d) of fruit storage (Fig. 6A). This pattern of *MaGBSSI-3* expression is consistent with the degradation of banana GBSS proteins during storage.

Starch granules were also observed in mature banana fruit using SEM after 0 d, 5 d, and 10 d of storage. Starch granules were detected at the first time point (0 d) and the number of granules decreased significantly thereafter. Fewer granules were observed at 5 d of storage and granules could not be detected by 10 d of storage (Fig. 6B). These results suggest that starch granules are degraded rapidly during storage of banana fruit and are also consistent with the pattern of *MaGBSSI-3* expression during storage of banana fruit.

## Discussion

### Changes in amylose content, GBSS enzyme activity, and GBSS protein

Amylose content is an important factor contributing to the yield and quality of banana fruit. Slack and Wainwright [18] reported that amylose content increased gradually during the development of barley grain, and the GBSS enzyme plays a key role in amylose synthesis [19]. In this study, changes in amylose content and GBSS enzyme activity were detected during banana fruit development and storage. Amylose content and GBSS enzyme activity increased gradually during the development of banana fruit but decreased significantly during storage (Fig. 1C and 1D; Fig. 1E and 1F). Regulating amylose content and GBSS enzyme activity is thus a promising method to enhance banana fruit yield and quality.

In our study, a GBSS protein purified from banana fruit was approximately 55.0 kDa (Fig. 2A and 2B). This molecular weight is similar to those GBSS proteins purified from rice (56 kDa) [20], maize (58 kDa) [21], and cowpea (58 kDa) [22]. SDS-PAGE and western blotting analyses indicated that banana GBSS proteins accumulated during the development of banana fruit and their levels decreased during fruit storage (Fig.2A and 2B). These results are consistent with the changes in amylose content and GBSS enzyme activity from banana fruit.

### Sequence analysis of *GBSS* genes

In this report, six *MaGBSS* genes were sequenced and characterized from banana fruit. *MaGBSSI-1*, *MaGBSSI-2*, *MdGBSSII-1* and *MaGBSSII-2* each have 13 exons and three conserved regions (Box1, 2, and 3) (Fig. S1 and Fig. S2). The structure of these banana *GBSS* genes is similar to that of *GBSS* genes characterized in rice [13], maize [12], sweet potato [8], amaranth [10], apple, peach, and orange [1]. We speculate that *MaGBSSII-2* is a pseudogene. In comparison to the other *MaGBBS* genes, this locus is characterized by frame shifts and stop codons in the first exon. *MaGBSSI-3* may be mainly responsible for GBSS activity in banana fruit since it is highly expressed in developing starch grains and is downregulated during starch degradation. *MaGBSSI-3* shares 100% sequence identity with a *GBSS* sequence fragment (accession No. AAQ06271) obtained from a SSH cDNA [23]. The *MaGBSSI-3* locus contains 6 exons and is characterized by 5 amino acid substitutions (T→S, F→M, P→F, C→Q, G→N) within Box 2 (Fig. S1 and Fig. S2). This gene has not been found in other species. In apple, three genes encoding GBSS, designated as *MdGBSSII-1*, *MdGBSSII-2*, and *MdGBSSII-3*, contain 13, 12, and 13 exons, respectively. Each of peach *PpGBSSII-1* and *PpGBSSII-2* contain 13 exons; orange CsGBSSII-1 and CsGBSSII-2 consist of 12 and 13 exons, respectively[1]. *GBSSI* genes from rice (AF141955), maize (NM001111531), wheat (AB019624), and barley (SBU23945) contain 13, 13, 11, and 11 exons, respectively. These results suggest that *MaGBSSI* might be the specific gene in banana fruit.

Genes encoding wheat *GBSSI* family members are located on chromosomes 7AS, 7DS, and 4AL [24]. The wheat *GBSSII* genes are located on chromosomes 2B, 2D, and on the long arm of chromosome 2A [11]. In this study, *MaGBSSI-1*, *MaGBSSI-2* and *MaGBSSI-3* were found on banana chromosome 1 and *MaGBSSI-4* is located on chromosome 9. Genes encoding banana *MaGBSSII-1* and *MaGBSSII-2* are located on chromosomes 4 and 8, respectively (Fig. S1). Thus, the localization of *MaGBSSI* and *MaGBSSII* genes to the different chromosomes suggest that *MaGBSSI* and *MaGBSSII* are encoded by separate genes.

### Expression of *GBSS* genes in banana tissues

The wheat *GBSSI* gene is exclusively expressed in reproductive tissues such as endosperm, embryos, and pollen, while the *GBSSII* gene is expressed in non-storage tissues including leaf, stem, root, and pericarp [11]. Within the eudicots, *GBSS* genes identified in apple, peach, and orange are expressed in both vegetative and reproductive tissues such as leaves, flowers, and fruits [1]. *GBSSI* in *Amaranthus cruentus* is expressed in storage-unrelated organs, such as leaf, stem, petiole, and root [10]. The pea *GBSSI* gene is expressed in leaves, pod, roots, and embryos, but not in flowers or stipules [25]. In our study, *MaGBSSI-1*, *MaGBSSI-2*, and *MaGBSSI-4* were upregulated expression in vegetative organs, such as root, stem, leaf, and bract (Fig.4). The expression pattern is similar to *GBSSI* genes in other eudicots [10], [25]. The banana *MaGBSSI-3* gene was abundantly expressed in reproductive tissues such as flower, peel, and pulp, while the *MaGBSSII-1* and *MaGBSSII-2* genes were highly expressed in vegetative organs such as root, stem, leaf and bract (Fig.4). The expression pattern of *GBSS* is more similar to *GBSSI* and *GBSSII* gene in monocots [11]. These results suggest that amylose accumulation in banana vegetative organs may be correlated with abundant expression of *MaGBSSI-1*, *MaGBSSI-2*, *MaGBSSI-4*, *MaGBSSII-1* and *MaGBSSII-2* in root, stem, leaf and bract (Fig.4). However, amylose accumulation in banana reproductive organs may require the activity of MaGBSSI-3 in flower, peel, and pulp (Fig. 4).

### Expression of *GBSS* genes at different stages of banana fruit development

In *Amaranthus cruentus*, GBSSI were strongly expressed in the middle and mid-late stages of seed development and decreased thereafter [10]. *GBSSI-1* expression in wheat endosperm may control starch synthesis at the post transcriptional level [5]. In apple fruit, *GBSSII* genes are highly expressed during all developmental stages. The *GBSSII-2* gene in peach is expressed only during the early development of the fruit and the *GBSSII-2* gene in orange is weakly expressed throughout fruit development [1]. The six *GBSS* genes in our study can be divided into two groups according to their temporal expression patterns. The early-expressing genes (*MaGBSSI-1*, *MaGBSSI-2*, *MaGBSSI-4*, *MaGBSSII-1*, and *MaGBSSII-2*) are expressed in the early stage (0–30 d) of starch granule formation, and are similar to the *GBSSII-2* gene in peach fruit [1]. The expression level of *MaGBSSI-1* and *MaGBSSI-2* in the early stages of banana fruit development was approximately 5-fold higher than that of *MaGBSSI-4*, *MaGBSSII-1*, and *MaGBSSII-2*. This result suggests that *MaGBSSI-1* and *MaGBSSI-2* might play an important role in early starch accumulation in banana fruit. The second group consists of a single, late-expressing gene (*MaGBSSI-3*), which is expressed in the later stage (30–60 d) of starch granule formation during the development of banana fruit (Fig.5A). These findings suggest that the six *MaGBSS* genes cloned from banana fruit contribute to starch accumulation at different stages of development and at varying levels.

### *MaGBSSI-3* might be involved in regulating the quantity and size of starch granules in banana fruit

In cereal mutants, debranching enzymes (principally isoamylases) have an effect on granule number and form [26]. Buleon et al. [27] reported that, in the GBSS -defective (sta2) mutant *Chlamydomonas reinhardtii* cells, starch granules are smaller (0.7–1.5 pm) and have more regular shapes. In potato tubers, SSIII activity also alters granule shape [14]. Slack and Wainwright [18] reported that small granules arise at an early stage of development within immature tissue, while large granules are found in mature tissue during the development of barley grain. However, the influence of GBSS activity on starch granules in banana fruit has not been reported yet. In this study, scanning electron microscopy analysis showed that this change in the quantity and size of starch granules was consistent with pattern of *MaGBSSI-3* expression during the development of banana fruit (Fig.6), suggesting that *MaGBSSI-3* might regulate the quantity and size of starch granules in banana fruit.

In conclusion, amylose content, GBSS enzyme activity, and GBSS protein levels gradually increased during banana fruit development, but they decreased during banana fruit storage. Full-length cDNAs encoding *MaGBSSI-1*, *MaGBSSI-2*, *MaGBSSI-3*, *MaGBSSI-4*, *MaGBSSII-1*, and *MaGBSSII-2* were 1851 bp, 1851 bp, 675 bp, 1845 bp, 2265 bp, and 906 bp, respectively. Among the six *MaGBSS* genes, only *MaGBSSI-3* was highly expressed in reproductive tissues and at the late developmental stages of banana fruit. Scanning electron microscopy analysis showed that the level of *MaGBSSI-3* expression is correlated with the quantity and size of starch granules in banana fruit during development and storage. These results suggest that up-regulated expression of *MaGBSSI-3* might be a key factor regulating amylose metabolism by affecting the variation of GBSS levels and the quantity and size of starch granules in banana fruit. Further work is required to elucidate how the different expression levels of the *MaGBSSI-3* gene at different times could result in changes of the quality and size of starch granules of banana fruit.

## Materials and Methods

### Plant materials

Banana (*M. acuminata* L. AAA group cv. Brazilian) fruits were obtained from the banana plantation at the Institute of Tropical Bioscience and Biotechnology (Chengmai, Hainan province, China). Root, stem, leaf, bract, flower, peel, and pulp tissues were collected separately using tweezers and frozen immediately in liquid nitrogen, then stored at −80°C until further analysis. Pulps of 0 d, 10 d, 20 d, 30 d, 40 d, 50 d, and 60 d after emergence from the pseudostem were collected, immediately frozen in liquid nitrogen and stored at −80°C for expression analysis.

Banana hands were separated into individual fingers representing the same developmental stage. The group of banana fingers was kept at 22°C and allowed to ripen naturally. In accordance with the banana ripening stages [28], fruits were incubated for 0 d, 5 d, 10 d, 15 d, 20 d, 25 d, and 30 d after harvest, then were frozen in liquid nitrogen and stored at −80°C until further analysis. All experiments were repeated three times.

### Determination of total starch content, amylose content, and GBSS enzyme activity

Banana pulp was immersed in 0.5% sodium bisulfite solution for 10 min to prevent browning, and then dried at 40°C for 24 h. Pulp was ground and centrifuged. The residue was suspended in 5 mL 80% Ca(NO_3_)_2_ in a boiling water bath for 10 min, and then centrifuged for 4 min at 4,000 rpm. The supernatant was transferred to a 20 mL volumetric flask, and the residue was extracted two times with 80% Ca(NO_3_)_2_, yielding a combined extract volume of 20 mL. All experiments were repeated three times. The total starch content was detected following the method of Yang et al. [29].

13 mg total starch was placed in a 10 mL graduated, stoppered test tube, to which was added 1.0 mL of 1M NaOH solution. The amylose content of banana pulp was determined following the method described by Yang et al. [29]. GBSS activity was detected according to the procedure of Nakamura et al. [24].

### Detection of GBSS protein levels by SDS-PAGE and western blotting

Starch granules were purified from 0.5 g banana pulp each sample, according to the method of Nakamura et al. [24] with some modifications. A 10 mg sample of purified starch was suspended in sample buffer (0.5 M Tris-HCL pH 6.8, 2.5% SDS, 10% glycerol, 2% 2-mercaptoethanol) and boiled for 3 min, then centrifuged at 15,000 rpm for 5 min. The supernatant was subjected to sodium dodecyl sulfate polyacrylamide gel electrophoresis (SDS-PAGE) using a 12% resolving gel and a 5% stacking gel. Gels were stained with Coomassie Blue and destained according to standard protocols.

GBSS protein samples were mixed with SDS-PAGE sample buffer, boiled 5 min, and separated on 12% polyacrylamide gels. Proteins were transferred to Hybond™ –N^+^ membranes (Amersham Biosciences, Buckinghamshire, United Kingdom) for western blot analysis. Membranes were probed with rabbit anti-GBSS polyclonal antibody (Abmart, Shanghai, China) diluted 1∶1000 in PBS-Tween 20 (PBST) plus 3% BSA, followed by alkaline phosphatase (AP)-conjugated anti-rabbit IgG secondary antibody (Sigma, Saint Louis, Missouri, USA) diluted 1∶1000. Positive signals on the membranes were detected by a 5-bromo-4chloro-3-indolyl-phosphate/nitro blue tetrazolium (BCIP/NBT) solution (Amresco, USA).

### Isolation of total RNA and synthesis of double-stranded cDNA

Total RNA was extracted from various tissues using the plant RNAout Kit (TIANDZ, Beijing, China). The integrity and concentration of isolated RNAs were examined by agarose gel electrophoresis and spectrophotometry (GelDoc-XR, Bio-RAD, Hercules, CA, USA). First-strand cDNA was synthesized in a 20.0 µL reaction mixture using 1.0 µg of total RNA, an Oligo(dT)_18_ primer and a Reverse Transcriptase M-MLV Kit according to the manufacturer's instructions (TaKaRa, Dalian, China).

### Cloning of six *GBSS* genes in banana fruit

GBSS homologues in banana were identified using a BLAST-based method. Primer pairs were designed to amplify each gene and their sequences are presented in Table S1. The PCR program consisted of 35 cycles of 40 s at 94°C, 40 s at 58°C, 90 s at 72°C, and a final extension for 10 min at 72°C.

PCR products were purified using an agarose gel DNA Purification Kit (TaKaRa, Dalian, China) and inserted into the pMD19-T vector (TaKaRa, Dalian, China). Ligated products were transformed into *E. coli* DH5α competent cells. Positive recombinant plasmids were selected by the white/blue selection method and verified by colony PCR. Target DNA was then confirmed by restriction enzyme digestion and sequence determination. Full-length cDNA sequences encoding *MaGBSSI-1*, *MaGBSSI-2*, *MaGBSSI-3*, *MaGBSSI-4*, *MaGBSSII-1*, and *MaGBSSII-2* were submitted to GenBank.

### Sequence analysis

Banana *GBSS* coding sequences were BLASTed against the banana genome sequence database (http://banana-genome.cirad.fr/) to recover their corresponding genomic DNA sequences. Exon lengths were calculated by alignment of genomic DNA sequences with cDNA sequences, and introns were determined according to the “GC-AG” rule [1].

Similarity of the full-length banana *GBSSI* or *GBSSII* cDNA sequences with other homologues in the GenBank database was performed using the BLAST program (*E*<0.001). The deduced amino acid sequences were aligned using the computer program Clustal W, and a homology tree was constructed with neighbor-joining method using MEGA software (Arizona State University, Tempe, AZ, USA). The number for each interior branch is the percent bootstrap values calculated from 1,000 replicates.

### Expression profiles of six *GBSS* genes in banana using quantitative reverse transcription PCR (Q-RTPCR)

Specific primer pairs were designed using Primer 5.0 software and their sequences are listed in Table S1. Expression levels of *GBSS* genes were quantified by Q-RTPCR using an iQ5 real-time PCR detection system (Bio-Rad, Hercules, CA, USA) and a SYBR *Ex*Script RT-PCR Kit (TaKaRa, Dalian, China). Each reaction of 25.0 µL contained 12.5 µL SYBR® Premix Ex*Taq*™ (TaKaRa, Dalian, China), 1.0 µL of each primer (5.0 µM), 8.5 µL ddH_2_O, and 2.0 µL cDNA (40 ng). An *actin* gene (accession No. EF672732) was used as an internal reference control.

The amplification program consisted of one cycle of 95°C for 1 min, followed by 40 cycles of 95°C for 10 s, 57°C for 15 s, and 72°C for 30 s. Melting curve analysis was performed at the end of 40 cycles to ensure proper amplification of target fragments. Fluorescence readings were collected from 60°C to 90°C at a heating rate of 0.5°C s^−1^ for melting curve analysis. Reaction mixtures lacking cDNA templates were run as negative controls to rule out contaminating DNA. All analyses were repeated three times using biological replicates. Relative expression levels of each gene were calculated using the 2^−ΔΔ*C*T^ method [30]. Data were analyzed using iQ5 software provided with the iQ5 real-time PCR detection system (Bio-Rad, Hercules, CA, USA).

### Scanning electron microscopy (SEM)

Based on the expression of *GBSS* genes during different stages of development and storage of banana fruit, pulp was isolated after 0 d, 30 d, and 60 d of development and from the fruit 0 d, 5 d, and 10 d following storage.

The samples were fixed in stubs using double-faced tape and coated with a 10 nm-thick platinum layer in a Bal-tec MEDo020 Coating system (Kettleshulme, UK). The samples were analyzed in an FEI Quanta 600 FEG Scanning Electron Microscope (FEI Company, Oregon, USA). SEM observations were performed in the secondary electron mode operating at 15 kV.

## Supporting Information

Figure S1 Sequence motifs and chromosomal localization of six genes encoding *GBSS* in banana. A, Structural organization of banana *GBSS* genes. Solid boxes indicate exons, and bold lines represent introns. B, Chromosomal localization of banana *GBSS* genes.
(TIF)

Figure S2 Multiple amino acid alignment of the complete coding sequences of MaGBSSI-1, MaGBSSI-2, MaGBSSI-3, MaGBSSI-4, MaGBSSII-1, and MaGBSSII-2 proteins from banana.
(DOC)

Table S1 Primers used in this study.
(DOC)
