# Supplementary figures and images for: Identification of Genes Encoding Granule-Bound Starch Synthase Involved in Amylose Metabolism in Banana Fruit
Source: PLoS One. 2014 Feb 4;9(2):e88077. doi: 10.1371/journal.pone.0088077 (PMC3913707; doi:10.1371/journal.pone.0088077)

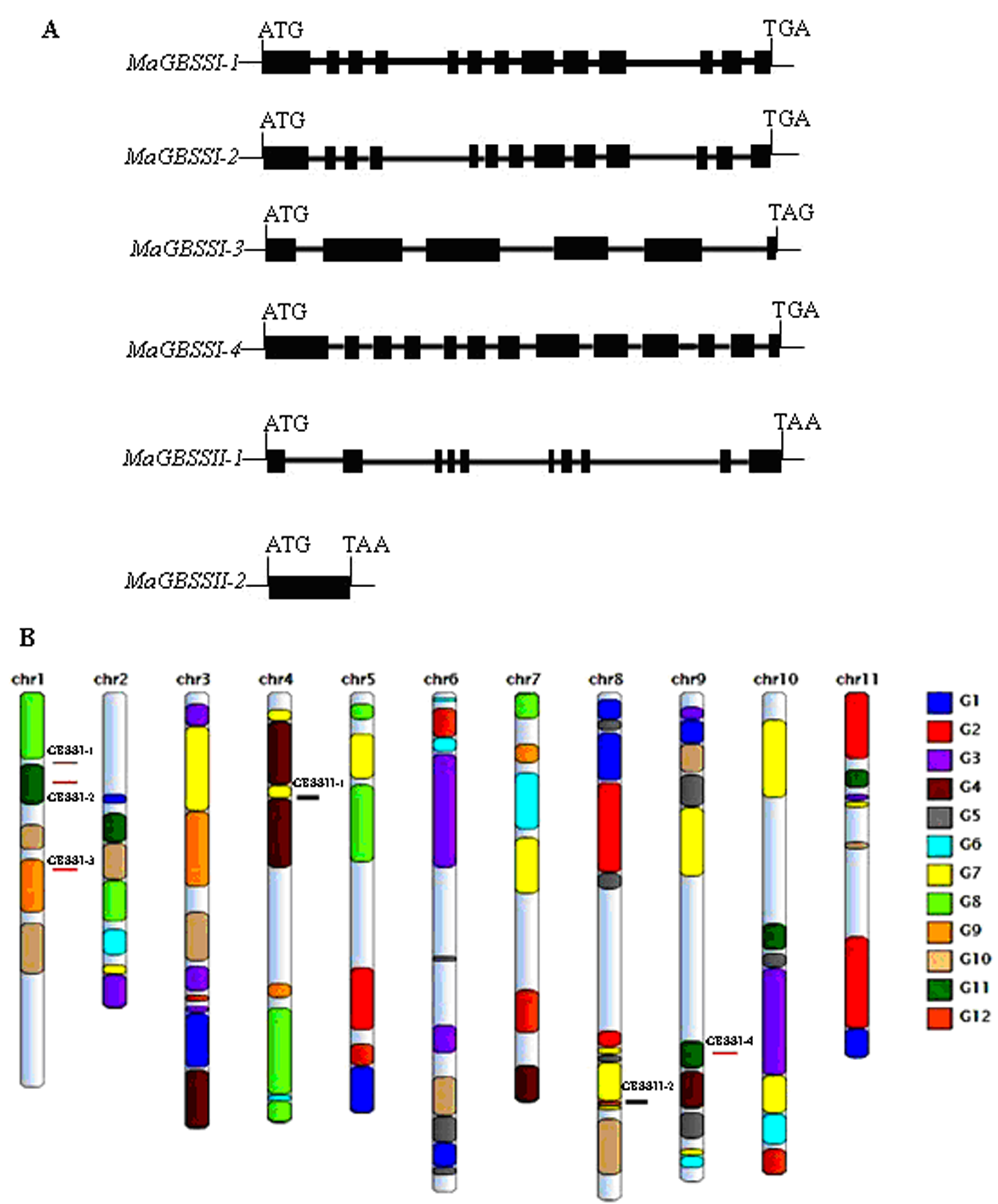

Supplement: Figure S1 — Sequence motifs and chromosomal localization of six genes encoding GBSS in banana. A, Structural organization of banana GBSS genes. Solid boxes indicate exons, and bold lines represent introns. B, Chromosomal localization of banana GBSS genes. (TIF) [file pone.0088077.s001.tif]
