# Supplementary material for: Identification of Genes Encoding Granule-Bound Starch Synthase Involved in Amylose Metabolism in Banana Fruit
Source: PLoS One. 2014 Feb 4;9(2):e88077. doi: 10.1371/journal.pone.0088077 (PMC3913707; doi:10.1371/journal.pone.0088077)
Supplement: Figure S2 — Multiple amino acid alignment of the complete coding sequences of MaGBSSI-1, MaGBSSI-2, MaGBSSI-3, MaGBSSI-4, MaGBSSII-1, and MaGBSSII-2 proteins from banana. (DOC) [file pone.0088077.s002.doc]

| MaGBSSI-1 --MAAVMASRFISRTSCSSYGGAFDSEAMTFQSRRVPYLSTHATTYEGLRTRNVVDSRQM |
| --- |
| MaGBSSI-2 --MAAVTKSQFISKGSCAGYGGVVDSEPRTFLNRRVLHLRNQTTAYEGLRSRNVVDLIQM |
| MaGBSSI-3 ------------------------------------------------------------ |
| MaGBSSI-4 --MATVTVLHFS-KGSCTSPGGFVDSEPRSFQSRRIPNSRNQITAYKGLRSENIVDSLRL |
| MaGBSSII-1 MSVPSTESKMSESRNSPMKTGESDDSLQSERLSPNPLGLAKDTDLDTTNPVEPIFLVKEP |
| MaGBSSII-2 ------------------------------------------------------------ |
|  |
| MaGBSSI-1 QLNAKATSRQARRGTRHASHRPWAIVVCGSG------------MNLVFVGAEVA**PWSKTG** |
| MaGBSSI-2 PSNAKVISRKTVRGTQNPSRRPWAVVICGKG------------MNIVFVGAEMA**PWSKTG** |
| MaGBSSI-3 ------------------------------------------------------------ |
| MaGBSSI-4 QSNAKATSTQAKTATRRASRRPLAVVVCGKG------------MNLVFVGAEMA**PWSKTG** |
| MaGBSSII-1 ERSVKDENAENQTEAKFDSVDPEADTDPEEENMDDPPLAGVNVMNVIVVAAECA**PWSKTG** |
| MaGBSSII-2 ------------------------------------------------------------ |
| **Box 1** |
| MaGBSSI-1 **GLGDVLGGLP**PAMAANGHRVMTVAPRYDQYKDGWDTG---VLVELKVGDRTETVRFFHCY |
| MaGBSSI-2 **GLGDVLGGLP**PAMAANGHRVMTIAPRYDQYKDGWDTN---VLAELKVGNEIERVRFFHCY |
| MaGBSSI-3 ------------------------------------------------------------ |
| MaGBSSI-4 **GLGDVLGGLP**PAMAANGHRVMTIAPRYDQYKDGWDTS---VQVELKVGNRVETVRFFHCY |
| MaGBSSII-1 **GLGDVAGALP**KALARRGHRVMVVVPRYGNYAEPKDTGTCFIQLLSYFGVQDMEVSYFHSY |
| MaGBSSII-2 ------------------------------------------------------------ |
|  |
| MaGBSSI-1 KRGVDRVFVDHPMFLEKVWGKTGGKIYGPVTGTDFEDNQLRFSLLCQAALEAPRVLHFNN |
| MaGBSSI-2 KRGVDRVFIDHPLFLEKVWGKTGGMIYGPVTGTDYEDNQLRFSLLCQAALEAPRILDLNN |
| MaGBSSI-3 ------------------------------------------------------------ |
| MaGBSSI-4 KRGVDRVFVDHPMFLAKVWGKTGGKIYGPATGTDYEDNQQRFSLFCQAALEAPRVLHLNN |
| MaGBSSII-1 IDGVDFVFVDHPVFQHQENNIYGGNRPDILK---------RMILFCKAAVEVPWHVPCGG |
| MaGBSSII-2 ------------------------------------------------------------ |
|  |
| MaGBSSI-1 SKYHSGPYGEDVVFIANDWHTALLPCYLKTMYQSHGIYKNAKVAFCIHNIAYQGRFAYSD |
| MaGBSSI-2 SRYYSGPYGEDVVFVANDWHTGPLPCYLKSMYQSHGIYKNARVAFCIHNISYQGRFAFSD |
| MaGBSSI-3 ------------------------------------------------------------ |
| MaGBSSI-4 SEYYSGPYGEDVVFIANDWHTGLLPCYLKSMHRSRGLYRNAKVALCIHNIVYQGRFALSD |
| MaGBSSII-1 VCYGDG----NLAFIANDWHTALLPVYLKAYYRDKGLMKYARSVLVIHNIAHQGRGPVED |
| MaGBSSII-2 ------------------------------------------------------------ |
|  |
| MaGBSSI-1 FARLNLPDKFKSSFDFIDGYDKPVKGRKINWMKAGIIESDRVLTVSPYYAQELVSGVEKG |
| MaGBSSI-2 FALLNLPDKFKSSFDFTDGSNRPVKGRKINWMKAGIIESDRVVTVSPYYAQELVSGVERG |
| MaGBSSI-3 ------------------------------------------------------------ |
| MaGBSSI-4 FALLNLPDEFKSSLDFTDGYDEPVKGRKINWMKAGIIESDRVVTVSPHYALELVG-EETG |
| MaGBSSII-1 FFHLDLPEKYMDLFTLYDPIG----GDHFNIFAAGLKTADRVVTVSHGYAWELKT-SQGG |
| MaGBSSII-2 ----------MDLFKLYEPMG----GDHFNIFAAGLKTADRVITVSRGYAWELTT-SEGG |
|  |
| MaGBSSI-1 VELDNILRMT--GITGIVNGMDTNEWNPSTDKYISAN----YDATTVMDAKPLNKEALQA |
| MaGBSSI-2 VELENALRMT--GITGIVNGMDTNEWNPSIDKYISVN----YDATNVMDAKPLNKEALQA |
| MaGBSSI-3 --------------------MDTNEWNPSTDRYISAN----YDATTVMDAKPFNKEALQV |
| MaGBSSI-4 VELDGVLRMT--GVTGIVNGMDVNEWNPSTDKYISTN----YDTATVMDAKPLNKEALQA |
| MaGBSSII-1 WGLHGIINDSDWKFRGIVNGIDIKDWNPELD------------------------AALQK |
| MaGBSSII-2 WGLHEIINENNWKFRGIVNGIDTVDWNPELDLHLQSDGYRNYSIETLQAGKPQCKAALQK |
|  |
| MaGBSSI-1 EVGLPVDPNIPVIAFIGRLEEQKGSDILASAIPEFIDEDVQ----VVVLGTGKKKLERQL |
| MaGBSSI-2 ELGLPVDRNIPVIAFIGRLEEQKGSDILAAAIPEFIDENVQ----VIVLGTGKKLLERQL |
| MaGBSSI-3 EVKLLVDRNIPVIAFIGRLEEQKGSDILAAAIPEFIDENVPSYSSLHLQGTGKKKLEQQL |
| MaGBSSI-4 EVGLPVDRNIPVVAFLGRLEEQKGSDIFAKAIPEFMDENVQ----VIVLGTGKKKLERQL |
| MaGBSSII-1 ELGLPVRRNVPVIGFIGRLDHQKGVDLIAEAMPWMVAQDVQ----LVMLGTGRPDLEEML |
| MaGBSSII-2 ELGLPVREDVPLIGFIGRLDHQKGVDLIAGAMPWIVGQDLQ----LVMLGTGRADLEEML |
|  |
| MaGBSSI-1 ALLETMFPDKVRAHLKFNVPLAHGIMAGADILAV**TSRFEPCG**LIQLQAMQYGIPPMC**STT** |
| MaGBSSI-2 AQLEDMFPNKVRAHMKFNAPLAHEIMAGADLLAL**TSRFEPCGL**IQLQGMRYGIPPMC**ATT** |
| MaGBSSI-3 ALLENMFPDKVRAHLKFNVPLAHGIMAACHSFCG**SSRMEFQNL**DHCENFLQ--PPMC**ATT** |
| MaGBSSI-4 AELEDMFPDKLRAHLKFNVPMAHAIMGGADLLAV**TSRFEPCGL**IQLQGMRYGIPAVC**STT** |
| MaGBSSII-1 RKFERENHDKVRGWVGFSVKMAHRITAGADILLM**PSRFEPCGL**NQLYAMKYGTVPVV**HAV** |
| MaGBSSII-2 RKFDREHHNKVRAWVGFSVKMAHRITAGADVLLM**PSRFEPCGL**NQLYAMKYGTVPVV**HAV** |
| **Box 2** |
| MaGBSSI-1 **GGLVDT**VKEGCTGFHMGPFSVECAVADKADVQKVVKTVKRALKVYG--TPAFAEMIQNCM |
| MaGBSSI-2 **GGLADT**VIEGITGFHMGPFNPDCDVVDKDDVQKVIQTVKRALKVYG--TPAFAEMIQNCM |
| MaGBSSI-3 **GGLVDT**VKEGITGLHMRPFNVDCDVVDEDDIQKVVKMTKRVLEVYQ--TAAFAKMIQNCM |
| MaGBSSI-4 **GGLVDT**VKEGITGFHMGRFSANCNVVDKEDIEKVVKTVKRAINVYR--TPAFAQMIQNCM |
| MaGBSSII-1 **GGLRDT**VVP----FDPFKETGYGWTFERAESGMLVHALGNCLNTYWNHKKSWEGLRTRGM |
| MaGBSSII-2 **GGLRDT**VIP----FDPFRESGFGWTFDRAEANKLINALGNCLNTYRNQKENWKGLQTRGM |
| **Box 3** |
| MaGBSSI-1 AQDLSWKGPAKKWEQFLLSLGAANSEPGIDGEEVAPLAVENVAAP |
| MaGBSSI-2 TQDLSWKGPAKKWEQFLLSLGAAGSEAGIDADEIAPLAKENVATP |
| MaGBSSI-3 DQDLSWKGPAKK--------------------------------- |
| MaGBSSI-4 KQDLSWKGPAKKWEQFLMSLGATGSEPGIDGEEIAPLAMENMATP |
| MaGBSSII-1 TQDLSWDNAAKHYEEVLVAAKYQW--------------------- |
| MaGBSSII-2 AQDLSWDNAAKHYEEVLVSAKYQW--------------------- |
