# Supplementary material for: Identification of Genes Encoding Granule-Bound Starch Synthase Involved in Amylose Metabolism in Banana Fruit
Source: PLoS One. 2014 Feb 4;9(2):e88077. doi: 10.1371/journal.pone.0088077 (PMC3913707; doi:10.1371/journal.pone.0088077)
Supplement: Table S1 — Primers used in this study. (DOC) [file pone.0088077.s003.doc]

**Table S1. Primers used in this study**

| **Gene name** | **Forward primer (5′-3′)** | **Reverse primer (5′-3′)** |
| --- | --- | --- |
| *MaGBSSI-1* | CCCATGGGATGGCTGCTGTGATG | GACTAGTCTCATGGAGCGGCCAC |
| *MaGBSSI-2* | CCCATGGGATGGCTGCTGTAACAAA | GACTAGTCTCAGGGAGTGGCCACATT |
| *MaGBSSI-3* | CCCATGGGATGGACACAAATGAGTG | GACTAGTCCTACTTCTTAGCTGGTCCCT |
| *MaGBSSI-4* | CCCATGGGATGGCGACTGTGACGGT | GACTAGTCTCACGGGGTGGCCATGT |
| *MaGBSSII-1* | CCCATGGGATGACGTCTTTCGGTTC | GACTAGTCTCACCATTGGTACTTG |
| *MaGBSSII-2* | CCCATGGGATGGACCTCTTCAAGCTG | GACTAGTCTTACCATTGATACTTGGCT |
| *MaGBSSI-1*-qPCR | CCTTCAGTGTGGAGTGTGCTGTTGC | ATTGCTCCCACTTCTTGGCAGGTC |
| *MaGBSSI-2*-qPCR | TGTCATAGAAGGCATCACAGGGT | CCACTTCTTAGCAGGTCCCTTCCA |
| *MaGBSSI-3*-qPCR | CACCACTGGCGGACTCGTTGA | GATCTTGGTCCATGCAGTTCT |
| *MaGBSSI-4*-qPCR | GTTGACAAGGAGGACATAGAAAAAG | TGGGCGAAGGCCGGAGTACGGTAG |
| *MaGBSSII-1*-qPCR | CGTCGTCCCCTTTGACCCCT | CAGATCCTGTGTCATCCCTC |
| *MaGBSSII-2*-qPCR | TCAGGGAGAGCGGGTTCGGGT | TACTTGGCTGAGACGAGGACT |
| *MaActin* | CAGTGGTCGTACAACTGGTAT | ATCCTCCAATCCAGACACTGT |
